# Neurotoxicity of Environmental Metal Toxicants: Special Issue

**DOI:** 10.3390/toxics10070382

**Published:** 2022-07-09

**Authors:** Richard Ortega, Asuncion Carmona

**Affiliations:** University of Bordeaux, CNRS, LP2I Bordeaux, UMR 5797, F-33170 Gradignan, France

Environmental exposure to metallic neurotoxicants is a matter of growing concern, since it may have very significant consequences for human health, from impairing neurodevelopment in children to the neurodegeneration processes involved in aging. The evaluation of the risks associated with the release of metals in the environment, the speciation analysis in environmental and biological samples, and the definition of relevant biological models to assess neurotoxicity are important research objectives. The aim of this Special Issue on the “Neurotoxicity of Environmental Metal Toxicants” is to provide a broad overview of the current work being performed in the field of the neurotoxicology of metallic contaminants, from the identification of emerging toxic compounds and the assessment of environmental exposures and associated risks to the description of the molecular mechanisms involved in neurotoxicity. Five original research articles and five review articles are reported in this Special Issue.

The impact of Alzheimer’s disease (AD) on human health is a matter of great concern. The causes of AD are still unknown and exposure to certain environmental metals may contribute to the etiology of this disease. In particular, the association of exposure to environmental arsenic and AD is an emerging field of research and the literature on this very important topic is reviewed by Rahman et al. [1]. Long-term exposure to arsenic has been associated with the loss of memory and of cognitive functions in humans, including early indicators of AD. Arsenic can induce oxidative stress, neuroinflammation, mitochondrial dysfunction, endoplasmic reticulum stress, and impaired calcium signaling. The authors also discuss possible prevention strategies such as using zinc or selenium supplementation.

The mechanisms of copper neurotoxicity and their presentation of commonalities with the known mechanisms of neurodegeneration in AD are reviewed in the article from Patel and Aschner [2], particularly concerning the relationship between beta-amyloid plaques and copper. Beta-amyloid plaques have two copper-binding sites that may be involved in the generation of reactive oxygen species. Copper ions have a high affinity for the metal-binding site of the Aβ peptide, which increases the proportions of β-sheet and α-helix structures in Aβ aggregations, thus forming plaques. In addition, exposure to excessive concentrations of copper may induce brain inflammation, which in combination with Aβ aggregation would contribute to the progression of AD.

There are multiple sources of exposure to environmental metals. One of these sources of exposure is food. The review article from Spencer and Palmer [3] examines the neurotoxic potential of metal/metalloids in plants and fungi used for food, dietary supplements, and herbal medicine. Some fungi are particularly likely to accumulate neurotoxic metals and metalloids such as arsenic, lead, cadmium, or mercury. Air pollution and industrial effluent discharges contaminated with metals lead to the accumulation of these metals in plants, and ultimately, in food. As reviewed by the authors, the neurotoxicity of metals through plant consumption can be direct or indirect by modulating chemical species known to produce neurotoxic effects.

Neurotoxic metals have a wide variety of intracellular targets in neurons where they alter physiological functions. One of the main targets of metal-induced neurotoxicity is the mitochondria, as shown in the review article from Cheng et al. [4]. Mitochondrial dysfunction has been associated with most neurodegenerative diseases, such as AD, Parkinson’s disease, Huntington’s disease, and amyotrophic lateral sclerosis, but also with autism. For example, arsenic-induced mitochondrial dysfunction in neurons is a central mechanism of neurotoxicity. The effects of arsenic, aluminum, copper, cadmium, mercury, lead, zinc, iron, and manganese on mitochondrial functions and their consequences on neurodegeneration are reviewed by the authors.

Synapses are another potential target of metal-induced neurotoxicity, as reviewed by Carmona et al. [5]. Neurotoxic metals can interact with neurotransmitter receptors, as is the case for arsenic, cadmium, manganese, and lead, through interactions with the expression or activity of several neurotransmitter receptors, mainly the glutamatergic receptors, but also dopamine, GABA, and acetylcholine receptors. Another molecular mechanism of metal-induced neurotoxicity is the modification of the synaptic structure targeting important scaffolding proteins such as SHANK3 or cytoskeleton proteins (e.g., F-actin and tubulin). The molecular mechanisms involved may be oxidative stress or competition with zinc- or copper-binding sites in synaptic scaffold and/or cytoskeleton proteins.

Two research articles reported in this Special Issue used the 2011–2014 National Health and Nutrition Examination Survey (NHANES) data. Barahona et al. determined the relationship between blood and urinary manganese levels and cognitive function using the NHANES data [6]. This is the first study to examine associations between blood and urinary manganese levels and cognitive function in an elderly population. Blood and urinary manganese levels were inversely associated with the CERAD (Consortium to Establish a Registry for Alzheimer’s Disease) after adjusting for all covariates; these associations were no longer significant after adjusting for the medical history. The findings suggest that increased blood and urinary manganese levels are associated with poorer cognitive function in an elderly population.

Metal neurotoxins not only affect cognition and memory but also neuromotor functions. This is well illustrated in the article from Gbemavo and Bouchard [7], who examined the relationship between blood concentrations of various metals and hand grip strength using the NHANES 2011–2014 dataset. They show that blood concentrations of mercury and manganese are not associated with grip strength, whereas lead is associated with a weaker grip strength in women, but not in men, even at low levels of exposure. In addition, low blood selenium levels were associated with weaker grip strength.

Methylmercury is an environmental toxicant of high concern, and chronic methylmercury exposure is associated with diabetes mellitus and metabolic syndrome. The study by Crawford et al. addressed methylmercury toxicity by examining its possible role in metabolic diseases using *C. elegans* [8]. The worms fed a cholesterol supplemented diet were more sensitive to methylmercury toxicity. The authors also examined whether the feeding habits of *C. elegans* were related to specific neuronal activity. The results showed that diet did not affect neurotoxicity pathways, such as dopaminergic dysfunction, in response to methylmercury exposure; however, diet did affect the feeding rate.

Another major concern in recent years has been cobalt-induced neurotoxicity after hip arthroplasty with cobalt chromium alloy prostheses. Gómez-Arnaiz et al. studied the mechanisms of cobalt neurotoxicity in rats treated with low concentrations of cobalt [9], which resulted in cobalt levels in the blood similar to those of patients with hip implants. A significant accumulation of cobalt in various organs of the rats, including the brain, was found. The authors measured the gene expression in neural tissue and revealed that the most up- or down-regulated genes were located in the choroid plexus, which is in direct contact with neurotoxicants at the blood–cerebrospinal fluid barrier.

Manganese is a well-known neurotoxic element, but the mechanisms of this toxicity are still poorly understood. Hernández et al. used SH-SY5Y neuroblastoma cells differentiated with retinoic acid to compare the toxicity of manganese chloride and manganese citrate [10]. Both chemical species of manganese induced similar toxicity governed by the disruption of protein metabolism, but with some differences. Manganese chloride impaired amino acid metabolism while manganese citrate inhibited the E3 ubiquitin ligase–target protein degradation pathway.

In conclusion, the scientific community will face many challenges in identifying and preventing the adverse effects of environmental metal exposure on brain health. We hope that this Special Issue will help to increase the visibility of this field of research, intensify collaborations, and increase the exchange of information between the different scientific communities concerned by this research topic.
